# Older Adults and the COVID-19 Pandemic, What About the Oldest Old? The PACOVID Population-Based Survey

**DOI:** 10.3389/fpsyt.2021.711583

**Published:** 2021-08-20

**Authors:** Virgilio Hernández-Ruiz, Céline Meillon, José-Alberto Avila-Funes, Valérie Bergua, Jean-François Dartigues, Michèle Koleck, Luc Letenneur, Camille Ouvrard, Karine Pérès, Nicole Rascle, Maturin Tabue-Teguo, Hélène Amieva

**Affiliations:** ^1^INSERM, Bordeaux Population Health Research Center, UMR 1219, Univ. Bordeaux, Bordeaux, France; ^2^Geriatrics Department, Instituto Nacional de Ciencias Médicas y Nutrición Salvador Zubirán, Mexico City, Mexico

**Keywords:** pandemic, lockdown, mental health, resilience, social-support, oldest-old adults

## Abstract

**Introduction:** The literature draws a mitigated picture of the psychosocial effects of the lockdown in older adults. However, the studies conducted so far are mainly based on web surveys which may involve selection bias. The PACOVID survey relies on a population-based design and addresses the attitudes, psychological and social experiences of the oldest old regarding the pandemic and lockdown and their impact.

**Material and Methods:** Cross-sectional phone survey involving 677 persons. Baseline report on attitudes, psychological, and social experiences of the oldest old, regarding the pandemic and lockdown measures.

**Results:** The mean age was 87.53 (SD 5.19). About 46% were living alone during the lockdown. Concerning difficulties, “*none*” was the most frequent answer (35.6%). For questions addressing how often they had felt sad, depressed, or lonely (CESD-scale), the most frequent answers were “*never/very rarely*” (58.7, 76.6, 60.8%) and 27.1% had anxious symptomatology (STAI scale). Most (92.9%) felt socially supported. Engaging in leisure activities was the most frequent coping strategy, and for numerous participants the lockdown did not represent much of a change in terms of daily routine. A very good knowledge and awareness of COVID-19 and the safety measures was observed. Comparisons with measures collected before the pandemic showed low changes in subjective health and the CES-D questions.

**Discussion:** With a methodological design limiting selection bias, our results claim for a weakened psychosocial impact even though the participants are concerned and aware of the pandemic issues. These results highlight the resources and resilience abilities of older persons including in advancing age.

## Introduction

Mortality rates due to COVID-19 clearly show that the older population is paying the heaviest tribute. The question of whether not only their physiological vulnerability but also specificities related to their psychological and social functioning contribute to this issue is not that clear. Whilst lockdown and social distancing have been one of the most recurred-to strategies since the first months of the pandemic, concerns about direct and indirect consequences of that measure have been raised, as the lockdown may impose a drastic change in daily activities and social interaction (1). Indeed, we know that older adults experience high rates of social isolation and that, apart from any crisis situation, socially isolated older adults present higher mortality and health-related events (2–5). Additionally, previous studies focusing on severe acute respiratory syndrome have reported increased suicide in older adults (6). Therefore, given the magnitude of the pandemic and the measures implemented by most countries resulting in limited contacts with friends and family, one can suspect strong impact on older persons. However, the impact may be more complex than it seems. Indeed, several studies show that older adults tend to have lower stress reactivity and better emotional regulation and well-being than younger adults (7, 8). Hence, recent data provide a mixed picture of how older adults are facing the pandemic. In a study involving 1,679 Dutch community-dwelling older adults (aged 65–102) who completed an on-line questionnaire, Van Tilburg et al. found that mental health measures remained roughly stable when comparing measures collected before and after the start of the pandemic, but also that loneliness had significantly increased (9). Czeisler et al. conducted a web-based survey in 5,412 community-dwelling adults recruited across the US. Among the participants, 933 were 65 or older and reported significantly lower rates of anxiety, depressive, or stress-related disorders than participants in the younger age groups (10). Similarly, Gonzalez-Sanguino et al. conducted an on-line survey in Spain involving 3,840 community-dwelling adults aged 18–80. The results show that older adults (60–80) compared with younger ones (40–59) presented lower rates of anxiety, depression, and post-traumatic stress disorder (11). Another on-line survey in a sample of 6,666 US adults assessed their perceived risks associated with the pandemic and completed a mental health assessment for anxiety and depression (12). They found that while the older group perceived higher risks of dying if getting COVID-19, they appeared to have a more optimistic outlook and had less depression and anxiety symptoms than younger participants. Finally, a cross-sectional study in the US and Canada involved 776 community-dwelling individuals who completed a daily diary tracing for positive and negative affect and stress symptoms during 7 days of the epidemic period (13). The results show those aged 60 and over compared with younger (18–39) and middle-aged adults (40–59) had less negative affect and more positive affect. Older adults also reported more positive daily events than the younger ones, despite similar level of perceived stress. Therefore, as Vahia et al. underline in their review, older adults as a group may be to a certain extent more resilient than the younger populations to the anxiety, depression, and stress-related mental health disorders of the COVID-19 pandemic, or at least during its initial phase (14).

However, there are important caveats to consider about the data published so far. One should keep in mind that a hallmark of aging is heterogeneity. Many older adults may not have the resources required to deal with the stress of COVID-19 due to economic (e.g., no access to digital communication tools), material (e.g., living in a narrow place with no garden), social (e.g., few relatives), or cognitive (e.g., inability to engage efficient coping strategies) issues. All but one of the previously mentioned studies consist in web-based surveys. While this method has obvious advantages as it allows collecting a substantial amount of data in large samples of participants in a short time, it also involves a major selection bias in particular when it comes to older population. Such a bias leads to over-representing within the study sample those older adults who are regular web users and have psychological and cognitive abilities to participate to the research. Such participants are less likely to experience the conditions that are suspected to increase the side effects of the pandemic on mental health as they may be younger, have higher education, higher income, and social status, and probably better physical, cognitive, and mental health. In older adults with cognitive impairment, the perception of the pandemic and the response to stress may be different as suggested by Di Santo et al., in their study involving older adults with varying degrees of cognitive impairment, an association was found between anxiety symptoms and the presence of subjective cognitive decline (15).

The PACOVID survey was set up in the region of Bordeaux (France) a few days after the first lockdown. It is based on a panel of participants who were already enrolled in three ongoing epidemiological studies on aging, in which they received regular follow-up visits at home. The population-based studies include a wide range of participants in terms of education, socio-economic status, living areas (rural/urban), and health status. As all participants were at least 80 years-old and over, the population was considered as part of the *oldest-old* age group. To facilitate the participation of the oldest persons, socially isolated individuals, those with bad health status, as well as participants who do not have access to the internet, the survey consisted in a two-wave telephone survey carried out during (wave 1), and after the lockdown (wave 2) addressing the following issues:

1. What are the attitudes, psychological, and social experiences of the older persons with regard to the COVID-19 crisis and the lockdown measures?
2. To what extent do such experiences have an impact on mortality and health events related and unrelated to COVID-19?

The present article reports the data referring to attitudes, psychological, and social experiences of older persons with regard to the pandemic and lockdown measures (i.e., level of stress, coping strategies, social support, access to digital communication tools, access to information, instructions, and measures put in place by government authorities, compliance to such measures) collected during the first wave of the survey, as the second goal of the PACOVID survey (i.e., impact on health and mortality) will be accomplished with further follow-up of participants.

## Materials and Methods

### Study Sample

The PACOVID survey was built in the framework of ongoing epidemiological studies on aging: PAQUID, 3-City, and AMI cohorts (16–18). Briefly, the PAQUID study is an epidemiological survey relying on a population-based sample of 3,777 community-dwelling individuals aged 65 or older randomly selected from electoral rolls. Participants were followed-up since 1988 until 2019 (16). The 3-City has been conducted in three French cities (Bordeaux, Dijon, and Montpellier). For the present study, only the Bordeaux sample is considered consisting of 2,104 community-dwelling individuals aged 65 or older randomly selected from electoral rolls, enrolled between 1999 and 2001 and followed-up until 2017 (17). Finally, AMI is an epidemiological study conducted to study the specificities of aging in rural communities. The initial sample included 1,002 retired farmers aged 65 and older who were randomly selected from the Farmer Health Insurance System. They were followed-up between 2007 and 2019 (18). For the three studies, the participants were evaluated at home approximately every 2 or 3-years. The clinical diagnosis of dementia was made following a three-step procedure: 1° a cognitive evaluation made by the neuropsychologist with a series of psychometric tests, 2° the participants who had a high likelihood of presenting dementia based on their neuropsychological performances were examined by a neurologist or geriatrician, 3°each case was discussed by a validation committee composed of senior neurologists and geriatricians to provide a consensual diagnosis.

For the PACOVID survey, the participants still followed-up within these studies were contacted by phone by trained psychologists and were invited to complete an interview. The first wave of the survey was conducted during the first lockdown (between March 11 and May 16) and the second wave 2–3 months after the lockdown.

### Collected Information

#### Previously Collected Information

Socio-demographics data [age, gender, and education considered in five categories (no or elementary education without diploma, elementary education validated by the primary school diploma, secondary level, long secondary level, university level)] were available in the ongoing cohort studies.

The Mini Mental State Examination (MMSE) score (19) as well as the diagnosis of dementia available at the previous follow-up visit were considered.

Previous information concerning subjective and mental-health measures, such as specific items from the CES-D scale (“*Have you felt sad/depressed/lonely*”) (20), and the STAI scale for anxiety symptoms were used to compare PACOVID measures to measures collected before the pandemic (21). For the CES-D questions, the answers provided by the participants at the previous follow-up visit (or when not available, at the preceding follow-up) were considered. Regarding the STAI scale, only the AMI cohort involved this measure, so a specific analysis on the AMI participants subsample was conducted to compare the scores collected during the PACOVID survey and those assessed before the pandemic.

The wave 1 of the PACOVID relied on a 45-min phone interview including the following questions:

- **Living conditions during the lockdown:**
  Participants were asked if they lived at their own home or if they lived in another place during the lockdown, and if so, for what reason. Did they have access to a garden/courtyard/balcony? Did they live alone? Did they benefit from home care/home support/home meal delivery services?
- **Coping strategies:**
  Participants were asked how they coped with the pandemic. They were free to give any answer. The answer was *a posteriori* classified by two independent raters. Thematic content analysis on the underlying type of coping strategy was performed with an inductive approach.
- **Mental health:**
  The short version of the STAI-state scale was administered to assess anxiety symptoms (score ranging from 10 to 40) (21). A cut-off score of 23 was considered for anxious symptomatology. In addition, three items from the Center for Epidemiological Studies Depression (CES-D) scale were administered: how often they had felt “*sad*,” “*depressed*,” and “*lonely*” during the past week. Each item is scored on a four-point scale (20).
- **Health status:**
  Participants were asked how they rated their health (very good/good/average/poor/very poor) and whether they had the following co-morbidities: diabetes, hypertension, stroke, heart failure, cancer, and respiratory diseases.
- **Functional status:**
  The six items of the Katz Activities of Daily Living scale (22) and five items (phone use, drugs management, domestic finances, shopping, transportation) of the Lawton and Brody's Instrumental ADL (IADL) scale (23) were administered. Instrumental ADL were not assessed for participants living in nursing homes.
- **Social support:**
  Participants were asked whether they had been contacted by the city council services during the lockdown; how many phone calls they received during the week (from family/friends/neighbors/professionals); how many visits they received during the week (from family/friends/neighbors/professionals); how many times they went out since the start of lockdown; from a general point of view, whether did felt supported during this period.
- **Digital tools use:**
  Participants were asked whether they had used digital tools to communicate with their relatives. If yes, how frequently? If no, would they have liked to use it?
- **Knowledge about the Covid-19 and the pandemic:**
  Participants were asked to provide the main symptoms of the COVID-19 (number of accurate and inaccurate symptoms provided was recorded). What are the persons for whom the pandemic could have severe consequences? What are the causes of the pandemic? (the participant was free to give any cause which was afterwards coded as “realistic” or “unrealistic”). What consequences could the pandemic have on themselves? (no consequence/minor/severe consequences). What could be the consequences for their relatives? (no consequence/minor/severe consequences). Did they feel well-informed? (yes/no).
- **Knowledge about the recommendations and policy measures taken in response to the pandemic:**
  Participants were asked to name the policy measures taken by the French government to fight the pandemic. Each correct measure provided was recorded. Did they think those measures were adequate? (yes/no). Did they think those measures could prevent them from catching the virus? (yes/no). Did they feel able to apply those protective measures? (not at all/a little/fairly/absolutely).
  A last question investigated whether they would vaccinate themselves if a vaccine were available (*yes, undoubtedly*/*maybe, later*/*no, I do not need it/no, I do not trust it*).

### Statistical Analyses

Descriptive analyses were conducted using frequencies and percentages for categorical data, and means and standard deviations (SD) for continuous data. Baseline characteristics of participants who completed the interview and those who did not complete it, and the measures collected before and during the pandemic were compared using χ^2^-tests, analyses of variance, and mean comparisons, as appropriate.

## Results

Responses to the questionnaire were obtained from a total of 677 persons. If the persons themselves could not respond to the questionnaire, a proxy, or a staff member for those living in nursing homes were invited to answer in their place, but only for specific parts of the survey which did not include subjective assessment such as difficulties during the lockdown, coping strategies, and self-perceived physical and mental health. Thus, 467 (69.0%) responses were directly collected from persons themselves (the *participants* group), 144 (21.3%) from a proxy, and 66 (9.7%) from the nursing home staff.

Baseline characteristics of the respondents who completed the interview (*n* = 677) and those who did not complete it (*n* = 142) were compared. Compared with the respondents, the non-respondents were not different according to age, gender, ADL, and IADL disabilities, or diagnosis of dementia. However, a difference in education (*p* = 0.0331) and MMSE score [25.04 (SD 4.76) vs. 23.88 (SD 5.53), *p* = 0.0216] between respondents and non-respondents was found considering the previous follow-up visits of the cohort study in which the participant is enrolled. Sample characteristics and comparison with non-respondents can be found in Supplementary Table 1.

Table 1 displays the sociodemographic characteristics of the participants. The mean age of the study population was 87.53 (SD 5.19), 192 (41.1%) participants were men, and almost half (47.3%) of the participants had elementary school education or no formal schooling. Additionally, 54.6% of this population had at least one comorbidity. At the previous follow-up visit, 2.8% of the community participants had a dementia diagnosis, 34.7% in the proxy group, and 65.2% in the nursing home group. Regarding functional status, 44.7% of the participants needed help for at least one IADL, and 39.2% for at least one ADL. A gradient in disability can be seen within the three groups of respondents (highest degree of disability in the persons living in nursing homes).

**Table 1:** Sociodemographic characteristics of the participants, PACOVID, *n* = 677.

	**All study sample**	**Participants**	**Proxy**	**Nursing home**
	**(*n* = 677)**	**(*n* = 467)**	**(*n* = 144)**	**(*n* = 66)**
Age, mean (SD)	88.88 (5.74)	87.53 (5.19)	90.60 (5.42)	94.66 (5.64)
Gender (men), *n* (%)	270 (39.9)	192 (41.1)	64 (44.4)	14 (21.2)
Education, *n* (%)				
No or elementary education without diploma	161 (23.8)	85 (18.2)	58 (40.3)	18 (27.3)
Elementary education validated by the primary school diploma	200 (29.5)	136 (29.1)	37 (25.7)	27 (40.9)
Secondary level	147 (21.7)	119 (25.5)	15 (10.4)	13 (19.7)
Long secondary level	79 (11.7)	58 (12.4)	15 (10.4)	6 (9.1)
University level	90 (13.3)	69 (14.8)	19 (13.2)	2 (3.0)
Last MMSE score available	25.04 (4.76)	26.67 (2.56)	22.87 (4.78)	18.24 (7.88)
Dementia, *n* (%)	106 (15.7)	13 (2.8)	50 (34.7)	43 (65.2)
ADL disability, *n* (%)	334 (49.3)	183 (39.2)	87 (60.4)	64 (97.0)
IADL disability, *n* (%)	319 (47.1)	201 (44.7)	118 (86.8)	
Comorbidities, *n* (%)	378 (55.8)	255 (54.6)	84 (58.3)	39 (59.1)

*ADL, activities of daily living; IADL, instrumental activities of daily living; MMSE, mini mental state examination; SD, standard deviation*.

### Living Conditions, and Coping Strategies During the Lockdown

The results revealed that 577 (95.5%) respondents for whom information was available reported that they stayed at their home during the lockdown, and 247 (45.7%) persons were living alone. Regarding the resources found at their homes, the majority (95.1%) reported having access to a balcony (24.9%), a hallway (42.9%), or a garden (74.7%) during the lockdown, while 33 respondents (4.9%) indicated not having access to any of them.

Participants' coping strategies, and perceived health during the lockdown period are presented in Table 2. Concerning the main difficulties faced during the lockdown, “*none*” was the most frequent answer (35.6%), followed by “*being in lockdown*” (29.3%), “*social isolation*” (23.1%), “*commodity supply*” (9.6%), “*worrying about people dear to them*” (9.0%), and “*being bored*” (6.6%).

**Table 2:** Coping strategies, and perceived health during the lockdown period, PACOVID, *n* = 467.

**Variables**	***n***	***n* (%)**
Difficulties during the confinement	467	
*Commodity supply*		45 (9.6)
*Social isolation*		108 (23.1)
*Being bored*		31 (6.6)
*Worrying about people dear to them*		42 (9.0)
*Worrying about them*		22 (4.7)
*Being in lockdown*		137 (29.3)
*Health status*		10 (2.1)
*Outdoor activity (leisure, sports, worship)*		13 (2.8)
*Stop/Reduction of medical or medical-Social care*		10 (2.1)
*Other*		12 (2.6)
*None*		166 (35.6)
Coping strategies	456	
*Distraction*		304 (66.7)
*Maintenance of daily activities*		111 (24.3)
*Compliance to safety measures*		91 (19.9)
*Acceptance of the lockdown situation*		68 (14.9)
*Engagement in healthy behaviors*		52 (11.4)
*Seeking social support*		28 (6.1)
*Non-compliance to safety measures*		14 (3.1)
*Seeking information*		9 (2.0)
*Expression of negative affect*		8 (1.7)
*Refusal of information*		5 (1.1)
*Religious coping*		4 (0.9)
Subjective health	458	
*Very good*		22 (4.8)
*Good*		249 (54.4)
*Average*		168 (36.7)
*Poor*		17 (3.7)
*Very poor*		2 (0.4)

The content analysis for the 456 answers provided to the question on how participants coped with the pandemic revealed that distraction (diverting attention doing leisure activities such as reading, watching television, playing games, gardening, doing crafts…) was the most common coping strategy (66.7%). Participants also mentioned that lockdown did not change much of their previous habits, so they coped with the pandemic period by simply maintaining their daily activities or routines (24.3%). Nearly 20% of the participants reacted to the situation by strictly observing the safety measures (barrier gestures, social isolation, organization for shopping…). Some participants described cognitive strategies of acceptance to the lockdown situation (14.9%), such as positive reinterpretation or adaptation to the situation. Engagement in healthy behaviors (e.g., indoor physical exercise) and seeking social support were, respectively, mentioned by 11.4 and 6.1% of the participants. Other coping strategies (non-compliance to safety measures, information seeking or refusal of information, expression of negative affect, and religious coping) were mentioned by a few participants.

For the 458 participants who reported how they perceived their health during the lockdown, the most frequent answers were “*good*” (54.4%) or “*average*” (36.7%). Only 4.8, 3.7, and 0.4%, respectively, answered “*very good*,” “*poor*,” and “*very poor*.”

### Mental Health, Social Support, and Digital Tools Use During the Lockdown

Data on psychological experiences, social support during the lockdown and use of digital tools are detailed in Table 3. For the three questions of the CES-D stating “*Have you have felt sad*,” “*Have felt depressed*,” and “*Have you felt lonely*” during the past week, the most frequent answers were “*Never/very rarely”* (58.7, 76.6, and 60.8%, respectively), and “*Occasionally*” (28.2, 13.8, and 19.4%, respectively). The answer “*Regularly*” represented 8.0, 6.5, and 9.4% of the responses, respectively.

**Table 3:** Mental health, social support, and digital tool use during the lockdown, participants of PACOVID, *n* = 467.

**Variables**	***N***	***n* (%) or** **mean (SD)**
Have you felt sad, *n* (%)	450	
*Never/Very rarely*		264 (58.7)
*Occasionally*		127 (28.2)
*Regularly*		36 (8.0)
*Frequently/All of the time*		23 (5.1)
Have you felt depressed, *n* (%)	449	
*Never/Very rarely*		344 (76.6)
*Occasionally*		62 (13.8)
*Regularly*		29 (6.5)
*Frequently/All of the time*		14 (3.1)
Have you felt lonely, *n* (%)	449	
*Never/Very rarely*		273 (60.8)
*Occasionally*		87 (19.4)
*Regularly*		42 (9.4)
*Frequently/All of the time*		47 (10.5)
STAI scale score, mean (SD)	417	18.69 (6.81)
High anxiety (score ≥23), *n* (%)		113 (27.1)
Number of phone contacts received, mean (SD)	441	13.65 (12.00)
Contacted by the city council services, *n* (%)	454	152 (33.5)
Number of weekly visits, mean (SD)	435	5.28 (7.07)
Perceived social support, *n* (%)	437	406 (92.9)
Digital tool use, *n* (%)	444	95 (21.4)

*SD, standard deviation; STAI, state-trait anxiety inventory*.

The mean score for the STAI scale assessing anxiety was 18.69 (SD 6.81). One hundred and thirteen participants (27.1%) had a score ≥23.

Regarding social support, for 441 respondents the mean number of phone contacts they received each week was of 13.65 (SD 12.00) during the lockdown. Also, one-third of the 454 respondents reported being contacted by the city council services during the lockdown, and 95 (21.4%) out of 444 respondents reported using a digital communication device. Despite the lockdown, a mean of 5.28 (SD 7.07) weekly visits were reported by 435 participants. Finally, 406 (92.9%) out of 437 respondents stated they felt supported during the pandemic.

### Knowledge About the Pandemic, the Recommendations, and Policy Measures Taken in Response

Table 4 presents the knowledge of the pandemic and safety measures. When asked about the possible consequences of the pandemic, 57.9% of the 440 respondents considered that the pandemic would entail “*severe consequences*” for themselves, whereas 18.0% responded “*minor consequences*,” and 11.1% responded “*no consequences*.” In contrast, when considering the potential consequences for their close relationships, 68.0% responded “*severe consequences*,” 13.5% “*minor consequences*,” and 2.5% “*no consequences*.”

**Table 4:** Knowledge and representations of the pandemic, and safety measures against COVID-19, participants of PACOVID, *n* = 467.

**Variables**	***n***	***n* (%) or** **mean (SD)**
Consequences of the pandemic, *n* (%)		
For themselves	440	
*No consequences*		49 (11.1)
*Minor consequences*		79 (18.0)
*Severe consequences*		255 (57.9)
*Do not know*		57 (13.0)
For their close relationships	438	
*No consequences*		11 (2.5)
*Minor consequences*		59 (13.5)
*Severe consequences*		298 (68.0)
*Do not know*		70 (16.0)
Number of correct symptoms, mean (SD)	414	2.69 (1.28)
Number of incorrect symptoms, mean (SD)	370	0.09 (0.31)
At least one realistic cause of the pandemic, *n* (%)	348	293 (84.2)
At least one unrealistic cause of the pandemic, *n* (%)	315	43 (13.7)
Knowledge the protective measures, *n* (%)	432	
*No*		22 (5.1)
*Yes*		389 (90.0)
*Do not know*		21 (4.9)
Ability to adopt the protective measures, *n* (%)	433	
*Not at all/quite a few*		8 (1.8)
*Fairly*		64 (14.8)
*Completely*		356 (82.2)
*Do not know*		5 (1.2)
If a vaccine against COVID-19 were already developed and available, would you take it? *n* (%)	449	
*Yes, without a doubt*		238 (53.0)
*Maybe, but not immediately*		120 (26.7)
*No, I do not trust it*		29 (6.5)
*No, I do not feel the need for it*		35 (7.8)
*Do not know*		27 (6.0)

*SD, standard deviation*.

When those respondents were asked to name the typical symptoms related to COVID-19, a mean of 2.69 (SD 1.28) correct symptoms were given, with extremely few (mean of 0.09; SD 0.31) incorrect symptoms mentioned.

When asked for the possible causes of the pandemic, 84.2% of 348 respondents mentioned at least one plausible or realistic cause (e.g., zoonosis), whilst 13.7% cited at least one unrealistic cause (e.g., divine punishment). Concerning the protective measures against COVID-19, 389 (90.0%) respondents answered that they knew the measures implemented by the French ministry of health. Of those respondents, the vast majority felt “*completely*” or “*fairly”* capable of adopting them (82.2 and 14.8%, respectively). Finally, when asked whether they would vaccinate themselves if a vaccine were available “*yes, without a doubt*” was the most frequent answer (53%), followed by “*maybe, but not immediately*” (26.7%), “*no, I do not feel the need for it*” (7.8%), and “*no, I do not trust it*” (6.5%).

### Comparisons Between Previous Follow-Up and PACOVID Results

Table 5 presents comparisons between subjective health, CES-D questions, and STAI score collected at the previous follow-up visits of the three original cohorts.

**Table 5:** Comparisons between subjective health, and mental health-related questions between previous follow-up, and PACOVID results.

**Variables**	**PA-COVID**		**COHORTS**		
	***N***	***n* (%) or** **mean (SD)**	***N***	***n* (%) or** **mean (SD)**	***p*-Value**
Subjective health	458		455		<0.0001
*Very good*		22 (4.8)		26 (5.7)	
*Good*		249 (54.4)		222 (48.8)	
*Average*		168 (36.7)		177 (38.9)	
*Poor*		17 (3.7)		26 (5.7)	
*Very poor*		2 (0.4)		4 (0.9)	
Have you felt sad, *n* (%)	450		449		0.0007
*Never/Very rarely*		264 (58.7)		319 (71.1)	
*Occasionally*		127 (28.2)		98 (21.8)	
*Regularly*		36 (8.0)		21 (4.7)	
*Frequently/All of the time*		23 (5.1)		11 (2.5)	
Have you felt depressed, *n* (%)	449		449		0.1784
*Never/Very rarely*		344 (76.6)		352 (78.4)	
*Occasionally*		62 (13.8)		71 (15.8)	
*Regularly*		29 (6.5)		18 (4.0)	
*Frequently/All of the time*		14 (3.1)		8 (1.8)	
Have you felt lonely, *n* (%)	449		449		<0.0001
*Never/Very rarely*		273 (60.8)		329 (73.3)	
*Occasionally*		87 (19.4)		54 (12.0)	
*Regularly*		42 (9.4)		43 (9.6)	
*Frequently/All of the time*		47 (10.5)		23 (5.1)	
**AMI subsample**	**216**				
STAI scale score, mean (SD)	191	17.51 (5.72)	177	12.53 (3.62)	<0.0001
High anxiety (score ≥23), *n* (%)		43 (22.5)		4 (2.26)	<0.0001

*SD, standard deviation; STAI, state-trait anxiety inventory*.

*The bold value corresponds to the N of the AMI subsample*.

For “*Subjective health*” evaluation, a significant difference between the measure assessed during the PACOVID survey and the one collected at the previous follow-up was observed (*p* < 0.0001) with less participants considering their health “*Poor*” or “*Very poor*” during the lockdown. Regarding depressive symptoms, significant differences were found for the questions “*Have you felt sad*” (*p* = 0.0007), and “*Have you felt lonely”* (*p* < *0.0*001), but not for the question “*Have you felt depressed*” (*p* = 0.1784). For the STAI scale, significant differences were found between the mean scores of AMI participants collected during the PACOVID survey and previous measures [17.51 (SD 5.72) vs. 12.53 (SD 3.62), *p* < 0.0001], and for the proportion of participants with scores ≥ 23 points (22.5 vs. 2.26%, *p* < 0.0001).

### Comparison in Mental Health Between Participants Living Alone vs. Not Living Alone

Supplementary Table 2 presents comparisons between the answers reported for the CES-D questions and the STAI scale by the participants who were living alone and those not living alone.

Statistically significant differences were observed for the different categories in all of the three CES-D questions (“*Have you felt sad*,” *p* = 0.0005, “*Have you felt depressed*,” *p* = 0.0032, “*Have you felt lonely*,” *p* < 0.0001). The category “*Never/very rarely*” remained the most frequently reported answer in both groups, but in lower proportions in participants living alone, as the “*Regularly*,” and “*Frequently/all of the time*” categories were more frequently reported in that group.

For the STAI scale, a statistically significant difference was observed between the mean scores [Living alone 19.8 (SD 7.5) vs. Not living alone 17.7 (SD 6.0), *p* = 0.0016]. No differences between the groups were observed for scores ≥23.

## Discussion

Older adults as a group, and more particularly those aged 85 and over, may be considered as particularly vulnerable in the context of the COVID-19 pandemic given their physical specificities resulting in increased risk for adverse health outcomes. However, not all individuals are equally vulnerable, including in very old age. The results of this study point toward a rather positive attitude, or at least a weakened impact on mental health, as some individuals who seemed to have little resources to deal with the burden generated by the pandemic showed remarkable adaptive abilities.

Most participants stayed at home during the lockdown, and nearly half were living alone. When asked for their main difficulties, “*none*,” “*being in lockdown*,” “*social isolation*,” and “*worrying about people dear to them*” were the most frequent answers. When asked for the potential consequences of the pandemic, the participants showed more concern for their relatives than for themselves. Regarding coping strategies, engaging in leisure activities was the most frequent one, and for numerous participants the lockdown period did not represent much of a change in terms of daily routine and activities.

In terms of self-perceived health, only a minority reported a “*poor*” health, and “*never/very-rarely*,” and “*occasionally*” were the most frequent answers for the questions on sadness, loneliness, and depression from the CES-D scale. Nonetheless, one on four participants presented significant symptoms of anxiety suggesting a real concern about the pandemic. Regarding social support, despite the safety measures limiting social contacts, nearly 90% felt supported during the pandemic. The mean number of weekly phone calls was around 13, and remarkably, nearly 21% had used a communication digital tool. These figures gathered from a population of oldest old individuals are encouraging for the promotion of digital devices in this population. Regarding the questions that addressed the COVID-19, most respondents correctly provided the typical symptoms, the plausible causes of the pandemic (a minority cited unrealistic causes), and the recommended protective measures. Remarkably, most participants showed a positive attitude toward vaccination.

Whereas, statistically significant differences were observed between PACOVID measures and those collected at the previous follow-up visits of the original cohorts, except for anxiety measures, they do not reveal a severe deterioration. Regarding “*Subjective health*” and the CES-D items, most answers remained “*Good/Average*” (*subjective health*) and “*Never/Very rarely* or *Occasionally*” (*felt sad/lonely*). For “*Subjective health*” evaluation, the answers “*Poor/Very poor health”* were even less frequent during the lockdown. For the anxiety score, however, the results reveal a slight increase in anxious symptoms with a five-point difference observed between the measures collected before and during the lockdown, as well as a higher proportion of participants with elevated anxiety scores in the AMI cohort. Finally, feelings of sadness, depression, and loneliness were more frequently reported by participants living alone. However, it is also remarkable that even in this group there was a non-negligible proportion of persons who reported not having experienced those feelings up to that point of the lockdown.

Such results may challenge the monolithic view of the older population as an extremely vulnerable group with respect to the lockdown and the pandemic when considering mental health. However, in the recent literature on the pandemic, results for both sides of the spectrum have been reported. The narrative review by Sepúlveda-Loyola et al. suggests a general negative effect in the older adult population (mainly based in communitarian non-institutionalized older persons) during the lockdown period. The review reports higher levels of stress, anxiety, depressive symptoms, and poorer sleep quality. This work relied on eight cross-sectional studies from diverse countries and showed that the prevalence for anxiety ranged from 8.3 to 49.7%, and for depression from 14.6 to 47.2%. Some of the observed risk factors associated with stress, anxiety, and depressive symptoms were being female, a negative self-perception of aging, lower familiar and personal resources, time devoted to COVID-19 information, having a close relation with a person with COVID-19, or previous medical problems (1, 24). The CHARIOT COVID-19 rapid response study is an online survey on 7,127 participants in the UK investigating the association between social isolation and mental and physical health of the older population. In their study, 5.5% presented anxiety symptoms, and 2.5% depression. The results also show an association between loneliness and a higher risk for reporting worsened levels of anxiety, and depressive symptoms following lockdown (25). Furthermore, in their study conducted in older adults with varying degrees of cognitive impairment, Di Santo et al. found an association between anxiety symptoms and subjective cognitive decline (15).

Contrasting with these results, some studies showed that when comparing mental-health related outcomes by age, it appears that the effect of lockdown due to COVID-19 is to some extent lower in older age. Several online surveys on community-dwelling older adults conducted in the US, Canada, Spain, and the UK, have reported lower rates of anxiety, depression, or stress-related disorder respect to younger participants (10, 11, 26). Other studies have reported less negative affect, more positive affect, and more positive daily events compared to younger adults, as well as a more optimistic outlook even while perceiving larger risks of dying if getting COVID-19 (12, 13).

Therefore, current literature draws a relatively mitigated picture of the psychosocial effects of the lockdown on the older adults, in which the consequences could be not that dramatic, except for certain groups, such as those with cognitive disorders or very socially isolated individuals.

Nonetheless, as most of the previous studies reporting a weakened impact on older adults relied on web-based surveys, one could suspect that the characteristics of the respondents could explain at least partly such results. Indeed, while online surveys have the advantage of assessing large samples in a short period, they involve important selection bias particularly in older persons, as the included population tends to be higher educated, healthier, and to have more resources (27, 28). With a different design, our study enriches the available literature on these issues as the interview was made by telephone by psychologists. This procedure allowed direct contact in a climate of confidence with the participants already enrolled in the ongoing studies, whose original design implied selection of the participants at random, hence, minimizing the selection bias. Indeed, usually underrepresented individuals contributed to the observed results, i.e., participants living in both rural and urban areas, almost half of the population had low education, 9.7% of the participants live in nursing homes, and among those persons a mean age of almost 95 years may be observed. Without such a design, it is far more than probable that most of these participants would not have participated in web-based surveys.

Interestingly, with a design limiting the selection bias, our results still claim for a weakened impact on mental-health measures even though the participants were well-informed of COVID-19 issues, and seriously concerned and aware of the potential consequences of the pandemic. This particular point potentially reflects older persons' lower stress reactivity. They may use emotional resources and also take benefit from the experience developed throughout their life to adapt in functional ways to face adverse situations (i.e., knowing the real implications of the situation without panicking); a finding which has also been observed during the initial confinement period by Novotny et al. (8, 29). Moreover, participants even showed a higher concern for consequences on close relationships than for themselves.

Our results suggest that the oldest-old are able to adapt and endure situations like the current pandemic. Indeed two-thirds of participants said that they used leisure activities to divert their attention from the lockdown and a quarter of them simply maintained their daily life habits or routines to cope with the pandemic situation. As oldest-old individuals generally present reduced mobility and only go out for specific reasons, the stay-at-home order may have had less impact than in younger populations who used to go out more frequently. Additionally, as suggested by van Tilburg et al., the potential negative effect of limited social interactions due to the lockdown period may have been countered by the effect of less (compared to younger adults) but very deep and meaningful relationships that continued during the pandemic (by different means like the telephone, or to a lesser extent digital tools) (9).

Other potential contributors for the low rates of distress could be the government programs that were developed in several countries, as soon as the lockdown started, as these programs specifically seek to contact the most vulnerable older persons of the community. French municipalities have started setting up these programs allowing the identification of isolated older adults after the heat wave in summer 2003, when the excess mortality had been very high in this population (30).

### Limitations and Strengths

Even though our study involves further follow-up, the data provided in this paper are cross-sectional. Moreover, despite that the sample size is not small for the study's design, it may limit some future statistical multi-adjusted analyses. The other main limitation is that the study does not allow comparing with younger age groups. However, several strengths can be underlined. This work only represents the first wave of a longitudinal study whose participants were already followed in different cohorts and come from diverse settings resulting in a diversified panel of participants. Moreover, given that the survey was based on phone interviews conducted by psychologists, it allowed direct contact with the participants and contributed to limit the selection bias. Finally, as already mentioned, the mean age of participants is almost 90, so it gives a relevant glimpse of the experience of the oldest-old population, commonly underrepresented within the literature.

## Conclusion

As challenging as the pandemic has been until now, and partly contrasting with the preconceptions one could have toward the older population, a growing number of studies, including ours, are highlighting the potential resources and resilience abilities of older persons including in advancing age.

This statement does not preclude better identifying among the older persons who are the more susceptible to develop negative consequences, as some conditions such as cognitive impairment, severe disability, or social isolation may act as important stressors in this context. Nor shall it preclude sustaining our efforts to continue studying the impact of the pandemic, since such mitigated impact in the first phase of the pandemic may worsen with time.

## Data Availability Statement

The datasets for the cohorts presented in this article are not readily available because they are property of the Université de Bordeaux. Requests to access the datasets should be directed to helene.amieva@u-bordeaux.fr.

## Ethics Statement

The participants have given their written consent to participate in the cohort studies. For the 3-City cohort, the study protocol was approved by the Ethics Committee of the University Hospital of Kremlin-Bicêtre, and participants signed informed consent (project number N°99-28). PAQUID (authorization number, CNIL 998-249) and AMI (registration number 2006-A00595-46) studies have received the approval from the ethics committee of the Bordeaux University Hospital according to the principles embodied in the Declaration of Helsinki. The patients/participants provided their written informed consent to participate in this study.

## Author Contributions

VH-R and HA designed the study, supervised the data analysis, and wrote the paper. CM performed statistical analyses and contributed to revising the manuscript. J-AA-F, VB, J-FD, MK, LL, CO, KP, NR, and MT-T revised the manuscript. HA designed the PACOVID survey. All authors contributed to the article and approved the submitted version.

## Conflict of Interest

The authors declare that the research was conducted in the absence of any commercial or financial relationships that could be construed as a potential conflict of interest.

## Publisher's Note

All claims expressed in this article are solely those of the authors and do not necessarily represent those of their affiliated organizations, or those of the publisher, the editors and the reviewers. Any product that may be evaluated in this article, or claim that may be made by its manufacturer, is not guaranteed or endorsed by the publisher.
