# Supplementary material for: Older Adults and the COVID-19 Pandemic, What About the Oldest Old? The PACOVID Population-Based Survey
Source: Front Psychiatry. 2021 Aug 20;12:711583. doi: 10.3389/fpsyt.2021.711583 (PMC8417796; doi:10.3389/fpsyt.2021.711583)
Supplement: Supplementary file 1 [file Data_Sheet_1.pdf]

Suppl. Table 1. Sample characteristics and comparison with non-respondents, n=819.

	n	Included n=677	Excluded n=142	*p*-value
Age, mean (SD)		88.88 (5.74)	89.79 (11.98)	0.3794
Gender (Men), n (%)		270 (39.9)	53 (37.3)	0.5707
Education, n (%)				0.0331
No or elementary education without diploma		161 (23.8)	40 (28.2)	
Elementary education validated by the primary school diploma		200 (29.5)	33 (23.2)	
Secondary level		147 (21.7)	33 (23.2)	
Long secondary level		79 (11.7)	26 (18.3)	
University level		90 (13.3)	10 (7.0)	
Last MMSE score available, mean (SD)		25.04 (4.76)	23.88 (5.53)	0.0216
ADL disability at last available follow-up, n (%)	787	94 (14.2)	23 (18.3)	0.2435
IADL disability at last available follow-up, n (%)	740	307 (46.5)	66 (52.8)	0.1970
Dementia, n (%)		106 (15.7)	31 (22.0)	0.0671

Note. ADL: Activities of Daily Living; IADL: Instrumental Activities of Daily Living; MMSE: Mini Mental State Examination; SD: Standard Deviation.

Suppl. Table 2. Comparisons in mental health between participants living alone vs not living alone of PACOVID, n=467.

		Living Alone	Living with other person(s)	
Variables	n	n (%) or mean (SD)	n (%) or mean (SD)	*p*-value
Have you felt sad, n (%)	448			0.0005
Never / very rarely		104 (48.6)	159 (68.0)	
Occasionally		73 (34.1)	54 (23.1)	
Regularly		23 (10.8)	13 (5.6)	
Frequently / all of the time		14 (6.5)	8 (3.4)	
Have you felt depressed, n (%)	447			0.0032
Never / very rarely		153 (71.8)	191 (81.6)	
Occasionally		29 (13.6)	32 (13.7)	
Regularly		20 (9.4)	9 (3.8)	
Frequently / all of the time		11 (5.2)	2 (0.9)	
Have you felt lonely, n (%)	447			<.0001
Never / very rarely		79 (37.1)	194 (82.9)	
Occasionally		64 (30.0)	23 (9.8)	
Regularly		31 (14.6)	10 (4.3)	
Frequently / all of the time		39 (18.3)	7 (3.0)	
STAI scale score, mean (SD)	417	19.8 (7.5)	17.7 (6.0)	0.0016
High anxiety (score≥23), n (%)		62 (31.3)	51 (23.5)	0.0742

Note. SD: Standard Deviation; STAI: State-Trait Anxiety Inventory.
