# Supplementary material for: Older Adults and the COVID-19 Pandemic, What About the Oldest Old? The PACOVID Population-Based Survey
Source: Front Psychiatry. 2021 Aug 20;12:711583. doi: 10.3389/fpsyt.2021.711583 (PMC8417796; doi:10.3389/fpsyt.2021.711583)
Supplement: Supplementary file 2 [file Data_Sheet_2.pdf]

Participant ID :

Cohort: Paquid / 3C / AMI

Date of the interview:

Interviewer: from 1 to 20

Respondent : Participant from a cohort / third person / nursing home staff

Refuses :

Date of birth : ______

If the participant cannot be interviewed mark the reason:

- Hospitalization
- Hearing problems
- Severe cognitive impairment
- Refuse
- Death
  - If yes, date of death: ____
  - Place:
    - Nursing home
    - Hospital
    - Domicile
  - City or name of the nursing home: ___________
  - Cause: ____________
- Other: _____________

To begin the questionnaire, while you think of the current pandemic, and the situation that you are personally experiencing, what are the three first words that come to your mind?

1st word __________

2nd word ___________

3rd word ___________

Living conditions during the lockdown period

1. During this lockdown period, are you living in your usual domicile: yes / no

If not, why?

- Not to be alone
- To live in a nicer environment / comfort (countryside, sea, garden…)
- To feel safer
- Be closer to shops / commerce
- Better medical services in case of disease
- Someone else took the decision for me
- Other: ___________

2. In the place that you are currently living, do you have access to a:

- A garden: yes / no
- A hallway: yes / no
- A balcony/terrace: yes /no

3. Are you living:

- In a nursing home:
- In a foster home:

o If living in a foster home, how many other older persons are living in the house: ___

- Home:

o If yes

□ Alone :

□ With partner / spouse :

□ With one or more other family members:

□ Other persons :

- In total, how many persons are living on the domicile? :___
- Are children present? yes / no
- Company animals / pets : yes / no

4. During the confinement have you had access to?

- Domiciliary nursing services: yes / no
- Domiciliary support services: yes / no
- Meal delivery service: yes / no
- Other professional service / other service: yes / no

5. Up to this point of the lockdown period what are the principal difficulties that you have experienced? (Open question)

- Commodity supply
- Social isolation
- Being bored
- Worrying about people dear to the
- Worrying about themselves
- Worrying about the country
- Being in lockdown
- None
- Other: __________

Question concerning coping

6. At your own manner, in what way do you face the current situation? What do you do?

Anxiety and depression

7. STAI scale, 10 items

	No	Rather not	Rather yes	yes
Have you felt tense, uptight				
Have you felt calm, good about yourself				

Have you felt moved, upset, aanoyed

Have you felt happy

Have you felt comfortable (do you feel good)

Have you felt nervous, irritable

Have you felt relaxed

Have you felt satisfied

Have you felt worried, concerned

Have you felt to not know where you stand anymore, disconcerted, confused

8. During the last week,

Have you felt sad?

• Never ; very rarely • ocassionaly • regularly • frequently ; all of the time

Have you felt depressed?

• Never ; very rarely • ocassionaly • regularly • frequently ; all of the time

Have you felt lonely?

• Never ; very rarely • ocassionaly • regularly • frequently ; all of the time

Health conditions during the lockdown period

9. Since the beginning of the lockdown, you would say that your health status has been:

Very good

Good

Regular

Bad

Very Bad

10. Have you had symptoms related to COVID-19? yes / no

11. Have you been diagnosed with COVID-19 (with a confirmatory test) ? yes / no

12. Does a person close to you has been diagnosed with COVID-19 (with a confirmatory test)? yes / no

13. Since the lockdown, have you had a medical problem (other than COVID-19) that required the advice of a health care professional? yes / no

14. If yes, which professional? :

o Attending physician: yes / no

o if yes, did he respond to your demand: rather yes / rather not

• Specialist : yes / no

o if yes, did he respond to your demand: rather yes / rather not

• SOS médecin (emergency home service): yes / no

o If yes, did he respond to your demand: rather yes / rather not

• Paramedics: yes / no

o If yes, did he respond to your demand: rather yes / rather not

• Nursing service : yes / no

o If yes, did he respond to your demand: rather yes / rather not

• Pharmacist: oui / non

o If yes, did he respond to your demand: rather yes / rather not

• Other: __________

15. ADL

Bath: (wash, basin, bath, shower)

0 ☐ Does not need help.

1 ☐ Needs help for one part of the body (back or legs or feet).

2 ☐ Needs help for bathing or diverse parts of the body / or impossible.

Dressing: (Takes clothing from the closet or drawers , including underwear and outerwear; uses buttons and zipper)

0 ☐ Does not need help.

1 ☐ Needs help only to tie shoes.

2 ☐ Needs help getting dressed or remains partially or completely undressed.

Going to the toilet: (To urinate or defecate, wiping and get dressed)

0 ☐ Does not need help (possible help to go to the toilet: cane, wheelchair ..., uses the basin).

1 ☐ Needs help.

2 ☐ Does not go to the toilet

Transfers:

0 ☐ Does not need assistance getting in and out of bed, sitting or standing from a chair (may use a support such as a cane or walker)

1 ☐ Needs help

2 ☐ Does not leave the bed.

Continence:

0 ☐ Complete control of urine and stool.

1 ☐ Occasional accidents

2 ☐ Total incontinence, need for catheterization or permanent monitoring.

Feeding:

0 ☐ Does not need help

1 ☐ Needs help cutting meat or buttering bread.

2 ☐ Full assistance or artificial feeding.

16. IADL

Ability to use the phone:

1 ☐ I use the phone on my own initiative, search and dial numbers etc...

2 ☐ I dial a few well-known numbers.

3 ☐ I answer the phone but do not call.

4 ☐ Unable to use the phone.

Shopping

1 ☐ I do all my shopping independently.

2 ☐ I only do small purchases on my own.

3 ☐ I need to be accompanied

4 ☐ Unable to shop.

Transport

1 ☐ I can travel alone and independently (by public transport, or with my own car).

2 ☐ I can travel alone by cab, not by bus.

3 ☐ I can take public transportation if I am accompanied.

4 ☐ Transportation limited to cab or car, accompanied.

5 ☐ Unable to use any transport

Medication:

1 ☐ I take care of the medicines myself: dosage and schedule.

2 ☐ I can take them on my own, if they are prepared and dosed in advance

3 ☐ Unable to take them by myself

Budget:

1 ☐ I am totally autonomous (managing the budget, writing checks, paying bills...).

2 ☐ I manage day-to-day expenses, but I need help managing my budget in the long term (to plan for big expenses).

3 ☐ I am unable to manage the money needed to pay my expenses on a daily basis.

Doubt about the reliability of ADL – IADL responses : yes / no

Health conditions before the COVID-19 crisis

Do you have any significant health problems? such as...

Diabetes yes / no

Hypertension yes / no

Ischemic heart disease yes/no

Stroke yes / no

Cancer yes / no

Chronic Lung disease yes / no

Other chronic disease : ____________

17. Have you had the flu in the last 6 months? yes / no

18. Have you had any other viral illnesses?

19. Vaccination related behaviors

20. Have you been vaccinated against the flu this year? yes / no

21. If a vaccine against COVID-19 were already available, would you like to receive it? yes without hesitation / maybe but not yet / no I don't trust / no I don't feel the need

Social support and use of digital tools

Since the lockdown, have the government services of your village / commune / city contacted you to ask how you are doing? yes / no

- How many days after the lockdown began? __
- Did this phone call reassure you? yes / no
- Did this phone call bring you useful information in the current situation? yes / no
- Did it result in the implementation of a specific service (e.g. meal delivery)? yes / no

22. Telephonic contact

22.1 Thinking about the past week (last 7 days), approximately how many phone calls have you received (excluding home professionals)? ____ (NUMBER)

- From a family member: yes / no

For what reason (open-ended question, but for this first question, read the possible answers as examples) (several answers possible): to check up on you / to distract you / to solve a particular problem, to do a service / to remind you of instructions / other

Do you currently receive more, less, or the same number of calls from them as usual? more calls / less calls / same as usual

- From a friend: yes / no

For what reason (open-ended question, but for this first question, read the possible answers as examples) (several answers possible): to check up on you / to distract you / to solve a particular problem, to do a service / to remind you of instructions / other

Do you currently receive more, less, or the same number of calls from them as usual? more calls / less calls / same as usual

- From a neighbor : yes / no

For what reason (open-ended question, but for this first question, read the possible answers as examples) (several answers possible): to check up on you / to distract you / to solve a particular problem, to do a service / to remind you of instructions / other

Do you currently receive more, less, or the same number of calls from them as usual? more calls / less calls / same as usual

22.2 Thinking of the past week (7 days), approximately how many phone calls have you made (excluding home-based professionals)? ____ (NUMBER

- Family member : yes / no

For what reason (open-ended question, but for this first question, read the possible answers as examples) (several answers possible): same options

Do you currently make more, less, or the same number of calls compared to the usual? more calls / less calls / same as usual

- Friend: yes / no

For what reason (open-ended question, but for this first question, read the possible answers as examples) (several answers possible): same options

Do you currently make more, less, or the same number of calls compared to the usual? more calls / less calls / same as usual

- Neighbor: yes / no

For what reason (open-ended question, but for this first question, read the possible answers as examples) (several answers possible): same options

Do you currently make more, less, or the same number of calls compared to the usual? more calls / less calls / same as usual

23. Physical contact

Still referring to the past week (last 7 days), how many visits did you receive?

- From a family member __
- From a friend: __
- From a neighbor: __
- Professional or domiciliary service: __
- Volunteer: __

Typically (outside of this crisis period), approximately how many visits do you receive (all visits combined) per week? __

24. Leaving your home

Since the confinement, have you gone out of your home to run errands (food or pharmacy...)? yes / no

If yes, how many times: ___

Usually, how many times a week do you go out for shopping? ___

25. Have you used other means, digital tools to communicate with your loved ones (skype, zoom, or other, any type of call with simultaneous vision of the person...)? yes / no

If yes :

How many times during the week: __

Do these calls help you through this crisis? yes / no

Compared to "traditional" phone calls, did they help you? More / Less / As much

If not :

In your opinion, would such tools help?

If you answered "no" :

Why? I don't feel able to use them / I don't see the point

26. Overall, do you feel supported during this period? yes / no

Knowledge and representations of the pandemic

27. To your knowledge / understanding, what are the symptoms of COVID-19?

(Potential symptoms: Fever, Cough and sore throat, Headache, Fatigue, Aches and pains, Respiratory discomfort, Loss of taste/smell, diarrhea)

Number of correct symptoms (0-8):

Number of other symptoms: __

28. For whom do you think this pandemic may have serious consequences?

• Everyone

• Babies

• Children

• Young adults

• Adults

• Pregnant women

• Older persons

• Persons with diseases

• Animals

• Others: _______________

29. Why do you think the pandemic has grown and continues to grow?

Number of realistic causes cited (e.g., globalization of trade, failure to comply with isolation orders, poor country preparedness for the crisis...) : __

Number of unrealistic causes cited (e.g., conspiracy theory, divine cause...): __

30. Do you think the epidemic will last? Yes / No

31. Could the pandemic impose consequences for you? no consequences / minor consequences / serious – severe consequences

32. Could the pandemic impose consequences for your loved ones no consequences / minor consequences / serious – severe consequences

33. How do you keep yourself informed of the current situation? State possible answers, multiple answers possible.

- Television : yes / no
- Radio : yes / no
- Printed media : yes / no
- Internet : yes / no
- People around you : yes / no
- I do not keep myself informed: yes / no

34. Overall, do you feel that the information you have is clear and sufficient?

35. Are you aware of the Ministry of Health's recommendations to fight the pandemic at the collective level? Yes / No

36. What are these official recommendations? Open-ended question; the aim is to check whether there is a good knowledge and understanding of:

- Physical measures : rather yes (if at least 2 barrier actions cited) / rather no / not cited
- Social distancing: rather yes / rather no / not cited
- Lockdown measures: rather yes / rather no / not cited

37. Do you think these measures are appropriate?

38. Do you think that applying these measures will protect you from the pandemic? rather yes / rather no

39. Do your relatives or people around you think that you should respect the measures? rather no / rather yes

40. Do you feel able to implement these measures? not at all able / somewhat able / quite able

41. Specifically;

Do you find the physical measures difficult to apply? rather yes / rather no

Do you find social distancing difficult to apply? rather yes / rather no

Do you find containment difficult to apply for you? rather yes / rather no

42. If yes to at least one of the 3 questions 41. What makes these measures difficult for you to implement? (open-ended question)

43. In general, do you think that there are other actions or other types of help than those proposed today by the town halls, the health professionals..., which would allow older people, to better live this situation? Yes / no

44. If yes, which :
